# Building Better Website Resources: What People Diagnosed with Sarcoma and Their Carers Want to Know

**DOI:** 10.3390/curroncol32120691

**Published:** 2025-12-08

**Authors:** Georgia K. B. Halkett, Jenny Davies, Chloe Maxwell-Smith, Connor Farnell, Mandy Basson, Tania Rice-Brading, Mariana S. Sousa, Janene Sproul, Helen M. De Jong, Haryana M. Dhillon, Joanna Elizabeth Fardell, Moira O’Connor

**Affiliations:** 1 Curtin School of Nursing, Faculty of Health Sciences, Curtin University, Perth, WA 6102, Australia; jenny.davies@curtin.edu.au (J.D.);; 2 Curtin Medical Research Institute, Faculty of Health Sciences, Curtin University, Perth, WA 6102, Australia; chloe.maxwell-smith@curtin.edu.au (C.M.-S.); m.oconnor@curtin.edu.au (M.O.); 3 Curtin School of Population Health, Curtin University, Perth, WA 6102, Australia; 4 The Abbie Basson Sarcoma Foundation Ltd. Sock it to Sarcoma!, Perth, WA 6906, Australia; mandy@sockittosarcoma.org.au; 5 Cooper Rice-Brading Foundation, Sydney, NSW 2011, Australia; taniarb@crbf.org.au; 6 Improving Palliative, Aged and Chronic Care through Clinical Research and Translation (IMPACCT), Faculty of Health, University of Technology Sydney, Ultimo, NSW 2007, Australia; mariana.sousa@uts.edu.au; 7 School of Education, Murdoch University, Perth, WA 6150, Australia; janene.sproul@murdoch.edu.au; 8 Curtin School of Allied Health, Curtin University, Perth, WA 6102, Australia; helen.dejong@curtin.edu.au; 9 Occupational Therapy, South Metropolitan Health Service, Fiona Stanley Fremantle Hospital Group, Perth, WA 6150, Australia; 10 Psycho-Oncology Cooperative Research Group (PoCoG), School of Psychology, Faculty of Science, The University of Sydney, Camperdown, NSW 2050, Australia; haryana.dhillon@sydney.edu.au; 11 School of Clinical Medicine, Faculty of Medicine and Health, University of New South Wales, Sydney, NSW 2033, Australia; j.fardell@unsw.edu.au

**Keywords:** sarcoma, patients, carers, healthcare professionals, qualitative research, website development, interviews, focus groups, survivorship care, rare cancer

## Abstract

People diagnosed with sarcoma and their carers often face significant unmet information needs that adversely affect their quality of life. A contributing factor is the limited availability of accessible, high-quality online information. This study aimed to determine the information needs of people affected by sarcoma from the perspectives of people with sarcoma, carers and healthcare professionals, to inform the development of web-based resources. People with sarcoma (n = 18), carers (n = 11), bereaved carers (n = 8) and healthcare professionals (n = 22) participated in interviews or focus groups (total N = 59). Data were analysed thematically. Nine themes were identified: “Accessing Useful Information About Diagnosis and Treatment”; “Learning to live with Sarcoma”; “Gaining Access to Psychosocial Support”; “Connecting with the Sarcoma Community”, “Obtaining Financial Support”; “Carer Self-Care”; “Facilitating Support for Family”; “Understanding Palliative Care”; and “Preparing for Bereavement and Coping After Death”. Findings support the development of a dedicated sarcoma website as a key step towards addressing their unmet needs. People with sarcoma and their carers highlighted that such a resource would not only improve access to reliable sarcoma-specific information, but also create opportunities for connection and shared experiences among individuals and families affected by sarcoma.

## Simple Summary

Sarcoma is a rare cancer, primarily affecting the soft tissue and bone. Limited online resources exist. This study aimed to determine the information needs of people affected by sarcoma from the perspectives of people with sarcoma, informal carers and healthcare professionals, to inform the development of web-based resources. People with sarcoma and their carers wanted information focused on diagnosis and treatment, living with sarcoma, accessing emotional support, connecting with others, obtaining financial support, palliative care, carer self-care, support for family and bereavement and coping. Further website resources need to be developed to address these needs.

## 1. Introduction

Sarcomas are a group of aggressive cancers, primarily affecting the soft tissue or bone [1]. Although rare, sarcomas represent 1.5% of adult cancers and 9% of pediatric cancers in Australia; the relative impact on individuals and the wider health economy is immense. Five-year relative survival rating is 69% for soft tissue sarcoma and 70% for bone sarcoma, much less than breast and prostate cancers [2].

Sarcoma has a significant impact on the physical and psychological well-being of individuals. As the disease progresses, individuals face ongoing pain, swelling, fatigue, nausea and limited mobility [3]. Treatment often comprises invasive surgical resections, involving limb salvage or amputation, chemotherapy, and radiotherapy. Such procedures can create functional issues, such as significant soft connective tissue loss, muscle and bone weakness, restricted range of motion, lymphoedema, neurological impairment, and uneven weight-bearing [4,5]. The traumatic nature of a sarcoma diagnosis, combined with its wide-ranging physical effects, often contributes to significant psychological distress [6].

High levels of stress, loss of hope for the future, isolation, and struggles with body image can lead to distress, anxiety, depression, and post-traumatic stress disorder [6,7]. Given the physical and mental burden of a sarcoma diagnosis, family carers play a pivotal role in an individual’s journey and require information and support from diagnosis through to survivorship or palliative care. Healthcare professionals (HCPs) who care for those with sarcoma face many challenges in supporting families, including managing the complexities of a rare cancer and providing targeted information for individual needs.

People with sarcoma and their carers report difficulty accessing credible information focused on sarcoma [7]. Our preliminary research identified gaps in providing information and support to people with sarcoma and carers across multiple domains, including a lack of information available about sarcoma, unaddressed practical concerns (e.g., how to manage daily activities, like showering after amputation), financial challenges (e.g., difficulties accessing government NDIS payments), and ongoing psychosocial issues [3,8]. These unmet needs led to feelings of uncertainty about treatment direction and disease prognosis, isolation, and difficulty functioning effectively in daily life [3,8].

Many of these gaps in supportive care could be addressed by providing additional information to people with sarcoma and their carers outside of primary healthcare settings. Seventy-seven percent of Australians diagnosed with cancer have used the internet to find more information about their diagnosis [9] and, when unable to gain information from their treating HCPs, they looked for information from community organizations and the internet [3,8]. There is increasing evidence that online resources can be effective in addressing individuals’ information needs due to their accessibility, wide reach, and sustainability [10]. Our review of available online sarcoma resources internationally highlighted a lack of reliable and accessible sarcoma-specific resources for individuals and carers [11]. This study, therefore, aimed to determine the information needs from the perspectives of people with sarcoma, carers and HCPs, to inform the development of an accessible web-based resource.

## 2. Materials and Methods

### 2.1. Study Design

An exploratory qualitative research design grounded in interpretivist epistemology and informed by symbolic interactionism was used [12]. Our approach focused on exploring how people affected by sarcoma perceive and describe their information needs. Symbolic interactionism was used to explore how individuals interpret and negotiate their information needs in social contexts [12]. This approach was used as social context has an impact on what information people are seeking and recognizes people are not accessing information in isolation [13]. Both semi-structured interviews and focus groups were used to collect data. Semi-structured interviews were used to allow individuals to describe their experiences and information needs over time. Health professionals had the opportunity to reflect on what information they provide and what information needs to be tailored, depending on individual’s situations and social context. Focus groups provided the opportunity for participants to discuss their information needs with others and construct shared understanding about information needs and resources needed to support people with sarcoma.

Ethics approval was obtained from Curtin University (HRE2023-0009).

### 2.2. Participants

Inclusion criteria included:

• People diagnosed and/or treated for sarcoma in the last 20 years.
• Carers, who have provided informal care to someone diagnosed with sarcoma in the last 20 years and included bereaved carers.
• HCPs who provide care to people with sarcoma.

Participants had to be 16 years or older and having sufficient self-reported English skills to participate in interviews. Participants who had previously participated in our earlier qualitative studies [3,8,14,15,16] were eligible to participate if they met the other inclusion criteria specified above.

### 2.3. Recruitment and Data Collection

Participants were recruited by expressing interest in the project via Sock it to Sarcoma!, the Cooper Rice-Brading Foundation—Australian sarcoma advocacy and support not-for-profit organizations, or social media posts by the investigators and community organizations on LinkedIn, X, Facebook, and Instagram. Recruitment flyers for HCPs were distributed via email, X and LinkedIn and through personal contacts at oncology treatment centres in Australia. Snowball sampling was used to expand the cohort.

Semi-structured interview/focus group guides for each participant group were developed. To ensure the quality of the participatory research process, the interview guide was reviewed by consumer representatives prior to use. Questions focused on understanding individuals’ and carers’ information and support needs, and perspectives on online sarcoma information. Follow-up prompts were developed to elicit detailed insights.

Online focus groups were conducted by two researchers (GH, MO) with each of the following groups: people with sarcoma, carers and bereaved carers. Semi-structured interviews were conducted by two members of the researcher team (GH, CF). It was not feasible to schedule any focus groups with HCPs due to their limited availability and our national recruitment. Semi-structured interviews focused on participants’ experiences and individual perspectives of information needs and allowed for an in-depth exploration of what new information resources people thought were needed. Semi-structured interviews were conducted with all participant groups until information power was achieved with no new information needs being identified [17,18].

Focus groups and interviews for all groups were conducted between 2 February 2023 and 22 May 2024 over the phone or via Microsoft Teams to enable participation nationally. Informed consent was obtained from each participant. Focus groups and interviews were digitally recorded and transcribed verbatim. Researchers conducting the focus groups and interviews reviewed the transcripts soon after the interviews, discussed results, and revised the discussion guides as required.

### 2.4. Data Analysis

NVivo Version 14 (Lumivero, formly QSR Internationally) was used to conduct thematic analysis. This was done in line with Braun and Clarkes’ steps of reflexive thematic analysis; familiarization with the data, generating initial codes, generating themes, reviewing and developing potential themes, refining, defining and naming themes, and producing the report [19].

### 2.5. Quality and Rigor

Several methods were used to ensure data transferability, credibility and dependability. Multiple researchers coded the transcribed data and developed initial themes (GH, CF, JD), ensuring a wide range of meanings were derived [20]. Feedback on the content and naming of themes was gained from all authors including investigators with lived experience caring for someone with sarcoma. An audit trail was maintained to document decision-making processes throughout data collection and analysis. Reflexive journaling was used to acknowledge and understand researcher bias [21]. The procedures and results of this study were reported using the consolidated criterion for reporting qualitative research checklist (COREQ) [22].

## 3. Results

Fifty-nine individuals participated in interviews/focus groups; people with sarcoma (n = 18), carers (n = 11), bereaved carers (n = 8), and HCPs (n = 22) (see Table 1 and Table 2 for demographics). Six focus groups were completed: three with people with sarcoma (n = 9), two with carers (n = 6) and one with bereaved carers (n = 3).

**Figure 1:**
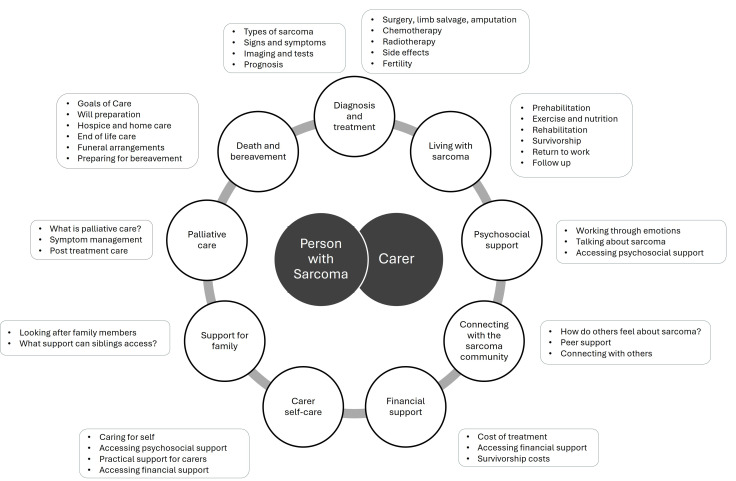
Summary of themes and examples of information needs relating to each theme.

The following themes were identified: “Accessing Useful Information About Diagnosis and Treatment”; “Learning to Live with Sarcoma”; “Gaining Access to Psychosocial Support”; “Connecting with the Sarcoma Community”; “Seeking Practical Support and Information”; “Obtaining Financial Support”; “Carer Self-Care”; “Facilitating Support for Family”; “Understanding Palliative Care”; and, “Preparing for Bereavement and Coping After Death”. Figure 1 summarizes the themes and provides examples of information needs relating to each theme. Abbreviations used for the interview exemplars include: PwS = person with sarcoma; C = informal carer of person with sarcoma; BC = bereaved carer of person with sarcoma; HCP = healthcare professional.

### 3.1. Accessing Useful Information About Diagnosis and Treatment

Participants were able to find some information of diagnosis and treatment readily available, but often information was presented in a way that heightened stress and uncertainty. Several participants described the negative information they found online, suggesting care should be taken with the framing of information provided:

*“Lots of information is very negative … for example, even if you put in Google and said how long does an amputee survive for? They’ve only got the five years …, you know, 5 years. Does that mean every amputee drops off?”* 

[PwS15]

There was insufficient Australian information on the internet about sarcoma.

*“Like the ones that my husband was Googling with international … They are available because they have a bigger population. There is obviously more people, but it’s very American centric and they have a lot more clinical trials and things going on over there.”* 

[C2]

Another, bereaved carer described a similar experience:

*“I’d search up Ewing sarcoma and it would just come up with American websites. And there just wasn’t anything in the online space.”* 

[BC2]

Participants described a need for sarcoma-specific information across a range of topics:

*“I was a patient so I’m thinking more information for patients … what is a sarcoma, types of sarcomas. The treatments that you can have, you’ve got your limb salvage surgery, you’ve got, the various, chemo and radiotherapy, possible drugs.”* 

[PwS1]

For carers, this lack of sarcoma information increased distress:

*“You feel like you get on this hamster wheel called ‘cancer’, and you just have to keep going. There was no time to explore options or to even understand what the word ‘sarcoma’ meant. Most people who are diagnosed with sarcoma have never heard of it before. Your world’s turned upside down, and you’re on this hamster wheel and just … trust the surgeons and the oncologists, the clinical people in front of you and you go with it. And that feels very disorientating and very disempowering.”* 

[C9]

Participants described HCPs as a key resource for information gathering; however, while the information provided was considered *high quality*, it was *not always readily available*. For some, this stemmed from short appointment times and a lack of opportunity to ask questions. One person with sarcoma noted:

*“I really had a lot of fear when I was first diagnosed. No-one really kind of sat down with us and said this is what it is. We found everything from Google … And it was really hard to find what I wanted to know.”* 

[PwS21]

People with sarcoma and carers reported a need for information that had high quality *and* high accessibility. Individuals also highlighted a lack of information pitched at an appropriate health literacy level:

*“I’ve got no qualms with how the medical team sort of provided us with information, but I feel like there is a big gap in that middle area, in between the sort of one-page summary of rhabdo [rhabdomyosarcoma] to the peer-reviewed academic articles, there’s nothing there.”* 

[BC2]

HCPs observed individuals and their families need comprehensive information given the complexity of the diagnosis:

*“Sarcoma patients need additional help and professionals aren’t adequate …. and anyone with a cancer diagnosis is, is fearful for their lives.”* 

[HCP8]

Some HCPs highlighted that health literacy plays an important role in an individual’s and a carer’s ability to understand online information:

*“If they get on Google and the internet, then they can get a whole realm of information that they might not necessarily be able to interpret or understand, and then that gives them anxiety.”* 

[HCP2]

Similarly, a person with sarcoma described encountering medical jargon: “*The websites coming up … were more medically based and statistics.” [PwS7]*, making it difficult for some to understand the information available.

Young people with sarcoma identified a need for information about fertility and discussed how this was managed:

*“I was told if you go ahead with freezing your eggs, it could be the difference between you seeing another sunrise or not.”* 

[PwS8]

For this participant it was explained that the time required to harvest and freeze eggs would delay treatment and may mean the disease would progress and treatment may no longer be curable.

Participants had quite differing health literacy levels and therefore differing information needs. Having access to information about clinical trials was important to individuals and their carers:

*“I’ve Googled PubMed, for research and things like that for different treatments and what trials are out there and that kind of thing. And at the moment I’m on a trial.”* 

[PwS21]

### 3.2. Learning to Live with Sarcoma

People living with sarcoma, along with their carers, described a wide range of ways they tried to adjust to life with the disease and cope with its impact. One patient recalled how she felt when she was told that her sarcoma diagnosis meant she would need a leg amputation:

*“It’s frightening. It’s sort of like saying to someone, I’m going to chop your hand off (or leg off) in 10 days. And you think, what do I do now? How do you mentally deal with that? Where? For me. Someone who loved to wear clothing, loved to wear high heels, makeup. And, do all that sort of stuff. I loved walking. I did it all the time. I had two dogs. I did that. I had to get my head around what was happening and sort it out.”* 

[PwS15]

People with sarcoma expressed how they lost their sense of control over their lives and the lack of answers to questions left them feeling powerless about their treatment:

*“Sometimes you feel like you’re on a train and instead of being in the seat next to the driver, you’re the passenger in the back and they’ve got the directions.”* 

[PwS8]

Some carers recognized the overwhelming experience for individuals with sarcoma and that they might not know what information they needed during clinical appointments:

*“I think she (the treating doctor) would answer every question I had and she was very responsive to what I wanted, but I think as a patient, you don’t know what information you want or need and your just feeling overwhelmed.”* 

[BC2]

Other people with sarcoma wanted guidance on looking after themselves and recommendations for before, during, and after treatment:

*“If I had access to more what I can and can’t do.”* 

[PwS21]

Individuals needed information on a wide range of practical aspects of prehabilitation, exercise and nutrition, rehabilitation, survivorship and returning to work. Some described practical information needs as small in contrast to their potentially life-threatening condition, but having a large impact on their well-being:

“It’s all about when you do find information, it will be about physical, psychosocial. But it’s not the boring, practical **** that you still have to get through.” 

[PwS9]

Carers of children needed practical information tailored to their children’s needs:

*“ And again, age-appropriate lists. Do you know what I mean? We don’t need any coloring-in books and pencils because she’s not going to do that … Just put a list of things for teenage kids, they’re so forgotten about.”* 

[C6]

People living in rural areas also highlighted that sometimes basic needs, like where to stay, became a challenge:

*“What? How do I get help? I don’t know anybody or I can’t stay with family. And travel is expensive. What do I access? Who do I go to for help? So even though we had family, we were not always convenient to stay. And particularly with some of the tests.”* 

[C8]

### 3.3. Gaining Access to Psychosocial Support

People with sarcoma and carers noted a distinction between medical support, focused on treating the physiological effects of sarcoma, and psychosocial support. Participants highlighted unmet psychosocial needs and explained that psychosocial impacts of the disease were ignored. For example, one carer described:

*“Certainly, in those early days when you, you just leave the hospital, you leave all your support network, and it’s all just medical support. There wasn’t a lot of emotional support, certainly not for me. Or for [family member with sarcoma] … you feel very vulnerable and isolated when you leave [the hospital].”* 

[C9]

Given the nature of sarcoma treatment and the significant impact on the individual and their carers, there is a need for information about psychosocial support and how to access it throughout this complex emotional experience:

*“I feel like some of the main [support needs], a lot of them are psychosocial because I think because of the way that sarcoma is treated, which is predominantly surgery, that’s not something you can really prepare yourself for with a diagnosis of sarcoma so something like psychosocial needs are really, really important when you get a diagnosis of sarcoma.”* 

[PwS1]

Participants wanted their HCPs to be aware of their psychosocial support needs, suggesting HCPs consider these when providing care:

*“What’s their [patients’] psychosocial, world looking like. And the support network. What’s that look like?”.* 

[PwS2]

HCPs also called for an increase in resources and communication about psychosocial support available for individuals and carers, with one oncologist stating,

*“In terms of the medical information and medical support and side effects, I think a lot of it is covered, maybe what’s not covered is what’s locally available in terms of psychosocial support.”* 

[HCP1]

Given the importance of psychosocial support and the gap in the provision of information about accessing psychosocial support by the healthcare system, there were requests for the development of informative resources and increased access to psychosocial supports for people with sarcoma and their carers.

### 3.4. Connecting with the Sarcoma Community

People with sarcoma, carers and bereaved carers reported a desire to connect with others. Social connectivity allowed timely and personalized access to information. Participants observed that questions would often arise outside scheduled healthcare appointments, leaving them without answers. Connecting with other people with sarcoma was seen as an answer to this problem: *“Just being able to connect and share stories of … a rare cancer … helped a lot.” [PwS21]* Peer support and connectivity were seen as pathways to emotional support and hope, and an important component of receiving cancer-related information. Given the rare nature of sarcoma subtypes, people with sarcoma commonly connect via online forums and social media, contextualizing the medical information they received and instilling optimism for the future:

*“… I was afraid of death. But I got through that fear and the fear that the doctors put on you like you’ve got to do this, and we’ve got to start it next week. And I think a lot of the Facebook groups that I like … they’re real people who have lived with this, some people lived with it for 20 years and you know, that gives me hope.”* 

[PwS5]

Most participants suggested that online services could improve outcomes for people with sarcoma and their carers. However, they also identified some risks with online peer support and connectedness:

*“I feel like if you’re linking with other people in that same space or going through the same thing, it can be a bit easier. But that’s a catch-22 because, I went on some random Facebook pages in that initial diagnosis, and I had to get off it because it was just rest in peace … Rest in peace …”.*

[PwS3]

Individuals and carers acknowledged the balance between gaining positivity and dealing with disheartening news. Participants explained that connecting with others as part of their search for treatment information via organizational sites had the potential to reduce negativity and foster hope:

*“So, I got in contact with her. And you know what? She was the one who’s told me that I’ll be fine … I needed to hear that. And she checked in with me … after the surgery and everything. And it was all through Instagram. Funny enough, it was all through [community organization]. And we have met now.”* 

[PwS15]

The benefit of connecting with others was a way to discover more information not discussed by their multi-disciplinary team, whether related to side effects of treatment or new international clinical trials. For example, many found benefits in gaining access to new information from other HCPs across the globe.

*“… being able to call on people in the UK or Italy or in the [USA] is hugely important, certainly for more unique sarcoma types, to tap into those other networks and bring those other opinions into the multidisciplinary discussion as well and not just necessarily rely on sarcoma centres in Australia.”* 

[PwS1]

Connection with other individuals with sarcoma provided the opportunity to discuss treatment options with others, empowering participants:

*“They’ve discussed it with their team, but they’re not sure, I wanna bounce it off another patient.”* 

[PwS17]

Carers felt it was important to connect with other carers and share insights; however, it was also important to have boundaries:

*“It feels like you become part of a different tribe and there’s something about connecting with that tribe that can be really helpful and supportive but I also know that you get a bit stuck in that cycle of trauma. I’ve done a lot of my own work in terms of navigating my own trauma. But certainly in those early days when you just leave hospital, you leave all your support network, and it’s all just medical support. There wasn’t a lot of emotional support … You feel very vulnerable and isolated when you leave.”* 

[C9]

### 3.5. Obtaining Financial Support

Participants described financial support needs throughout their sarcoma journey. One carer described the lack of information regarding financial support and the value of easier access to such support:

*“In respect for financial support for people in our situation [working part-time]. But while studying and looking out for the kids and looking after my husband and trying to get him to all of his appointments, it’s just … full on.”* 

[BC3]

HCPs noted the need for more financial information for families who have been impacted by sarcoma:

*“And then adults unfortunately do get cancer and I think the most vulnerable time for our patients is between 25 and 50 to tell you the truth because that’s when patients are trying to work and trying to make money and having to be independent and so that’s probably the biggest gap, for me is that additional support and for most part that’s financial.”* 

[HCP8]

For carers, access to online information on funding was a key unmet need. The financial pressure resulting from a sarcoma diagnosis is compounded by balancing their role as a carer for their family and the time taken for this role. One carer described that, while they need financial assistance, much of their time is spent caring for and supporting their loved ones, meaning accessing financial support was difficult:

*“… I’m on my daughter’s bedside. She’s literally like taking four forms of nausea medication. I can’t leave her for one second to go and, register at Centrelink… Otherwise, we can’t help you. So, I still to this day, we’ve never received a cent …”* 

[C4]

One participant described creating a checklist as a helpful method of helping others consider different funding options and to help with managing information:

*“So, check your Super (annuation). Do you have options within that financially or these are some of the things you might consider, I had not considered any of them. All I had thought in terms of finances was I’ll go public because I can’t afford to go private … I can’t work, so even if it’s that checklist of not necessarily, here’s the person that you call …”* 

[PwS9]

For others, a lack of financial support impacted individuals and families during survivorship. Individuals need funding for rehabilitation and access to Australia’s National Disability Insurance Scheme (NDIS) is a prevalent concern for many. One healthcare professional explained:

*“Financial things are probably the most important things and what government support is available for them. And also, for the longer-term sarcoma patients, how they access the NDIS and other services if they’re left with a significant disability.”* 

[HCP6]

Changing occupational roles was a financial barrier that individuals and carers had not previously considered needing support with. One HCP explained:

*“… A lot of those guys that are diagnosed in their fifties, and if it’s a big resection, are probably going to have to change careers or retire early and so that can be a struggle for them.”* 

[HCP6]

Inadequate provision of financial guidance contributed to unmet information needs for individuals with sarcoma and their families.

### 3.6. Carer Self-Care

A common characteristic among carers was a focus on seeking additional information to provide care and support to others. At times, carers would neglect their own physical and mental health as they were focused on their caring role:

*“The parent ignores themselves completely and just puts the child first and everything else and they push themselves and push themselves and push themselves and then they fall off the precipice.”* 

[C1]

The carer’s role was complicated and involved many tasks necessitating information seeking, including coordinating and attending diagnostic tests and appointments, as well as organising finances:

*“We were sent off for more scans and more scans … constantly on the phone having to rattle cages about … Do you really need this scan? or, What about his prosthetic? or NDIS takes six months, and two ministerial complaints and an ombudsman complaint to even give him any money for rehab, or having to follow up with Centrelink to try and get him some financial support. All of those things on top of having a child that could be dead in a couple of months, it’s too much.”* 

[C2]

Carers and HCPs desired supports and educational resources to take better care of themselves:

*“I think carers need carer support in the way of having a bit of a reprieve themselves … and also, education on their well-being, personal mental health well-being. I think carers don’t have a good sense that I need to make sure I’m okay in order to do my job well.”* 

[HCP3]

Self-care was seen as a way for carers to look after their own physical and mental health. For example, communicating with other carers in similar circumstances was considered helpful:

*“I just needed somebody who understood what we’re going through as parents.”* 

[C4]

Carers identified the importance of reminding new carers it is OK to be ‘selfish’ and providing lived experience insights to others:

*“Let them know it’s okay that they might need to have some time for themselves and that it’s not being selfish to do that; it is helping the person they’re looking after because they’re in a better space to do that.”* 

[C9]

### 3.7. Facilitating Support for Family

This theme highlights the dilemma of supporting and providing resources to other family members while families’ and carers’ attention was focused on the person with sarcoma. Many recognized the need to support other family members, including siblings, and the difficulties doing so:

*“I often referred to him [sibling] as my forgotten child, because that was around the guilt that I felt.”* 

[C2]

Carers required external assistance to provide care for other family members.

*“But I think, from the moment, someone in the family is diagnosed with cancer, the whole family need to be taken care of as a whole; not just the patient, but the family.”* 

[C3]

Participants expressed that the information and support needs of their other family members were not recognized by the healthcare system, leaving the onus on the carer(s):

*“… we were never offered anything for any of them … my other two little ones take after me, so they have anxiety as well. And so, we see a counsellor for them anyway.”* 

[C4]

### 3.8. Understanding Palliative Care

This theme identified a knowledge gap regarding palliative care: “*I was just desperate for information [about palliative care]” [BC2]*. For some, this lack of information compounded their already high level of anxiety and distress. Calls for a clear definition of palliative care were highlighted. It was implied the common misconception that palliative care equates to death, which is not the case:

*“I think when people get referred to palliative care, they freak out because they think hospitals think they’re dying—it’d be good if there was a clear definition of what palliative care is and that you know if you get referred to palliative care services, it doesn’t actually mean that you’re gonna die.”* 

[BC2]

There seemed to be a lack of information on palliative care for family members. One sibling reflected not getting any information on palliative care

*“ But just what palliative care looks like, we didn’t really get information on. Maybe my dad did. But, as adult siblings, we would have probably liked to aswell.”*

[BC5]

Carers also commented on the training HCPs receive on providing palliative care information, suggesting further specialized training, particularly communication skills, would be beneficial to sensitively address information needs:

*“You’re studying palliative care or you’re studying end-of-life for a patient, how do you tell a patient they’re going to die and what are you going to have in place to support them, as a doctor? I don’t even reckon that’s probably ever studied.”* 

[C13]

### 3.9. Preparing for Bereavement and Coping After Death

Families expressed the need for ongoing education and support after a child dies from sarcoma:

*“And then, bereavement support for those families whose children don’t survive. I think there needs to be quite a broad spectrum of support.”* 

[C10]

Information regarding bereavement, grief and coping after death was needed:

*“… once you move out of the system with a bereavement there is a huge gap and people feel very unsupported.”* 

[BC7]

Bereaved carers wanted information to cope with death and grieve: *“I was probably more searching for information around … grief” [BC5].* This need occurred for bereaved carers and family members:

*“I know that (psychological support) would have been a real benefit and I think it would have helped them [family members] with some of their grieving.”* 

[BC6]

While some were looking for information on the practicalities of death, carers believed speaking with others could support them to cope with the passing of a loved one:

*“Her experience is something that I really would have benefited from. I really wanted to talk to other parents of kids going through rhabdo [rhabdomyosarcoma].”* 

[BC2]

Bereaved carers highlighted that while information needs to be accessible, people may not want to receive all information at once. Therefore, information on the website needs to be structured appropriately:

*“It’s like a one step at a time thing and to see how to plan a funeral when you’ve just been diagnosed is just like a step, way, way, way, too far. So, you do need to be careful. I think as to where you place that information.”* 

[BC4]

## 4. Discussion

Given its rarity and significant impact on daily functioning, sarcoma diagnoses pose unique challenges for people with sarcoma, carers, and HCPs. We determined the information needs of people affected by sarcoma from the perspectives of people with sarcoma, carers, and HCPs to inform the development of web-based resources.

Like people with other rare cancers, people with sarcoma and carers in this study tended to independently seek information online [23], yielding limited information focused on the prognosis of sarcoma, with inadequate information on supportive care topics. Unfortunately, this experience is not isolated to our cohort. In patient surveys in the UK, 45% of people with sarcoma reported information available to them was not adequate at diagnosis [24]. People with sarcoma and carers report a lack of accessible medical information about treatment options, side effects, prognosis, recovery and bereavement [3,8,14,25,26]. Findings in this study add to this growing evidence of the limited information regarding treatment information, carers’ needs, psychosocial support, practical and financial support, support for family, palliative care and bereavement. A lack of information seemed to exacerbate feelings of powerlessness, distress, and anxiety in people with sarcoma and their carers.

Despite their central role in care provision, carers and family members receive less support than people receiving sarcoma treatment, despite having similar or higher levels of distress [27]. Carers report difficulty finding time to care for themselves, possibly due to the perception of their role as facilitating, rather than receiving, care [28]. However, participants desired education about the impact of carer burnout, along with connection with other carers. Social connection has been posited as a buffer to the negative effects associated with the carers role (e.g., loneliness, isolation, burnout) [29], and should be an important consideration in the development of online information and support resources.

Bereaved carers required information on the management of bereavement issues, for example, anticipated timeframes of end-of-life stages and having conversations with people with sarcoma at the end of life. Upon the death of a person with sarcoma, the needs of bereaved carers increased, while the level of support was perceived to decrease. The death of a loved one signaled *“moving out of the system”*, indicating heightened risk for the bereaved carer and family’s well-being. Healthcare support for bereaved carers and families has been reported as sporadic [30], warranting a better understanding of bereaved carers’ needs to develop appropriate interventions at critical periods.

### 4.1. Limitations

The recruited cohort represented English speaking people from a Caucasian background. Given the likelihood of specific needs of Indigenous Australians, as well as those who speak English as a second language, the needs of these groups should be explored. While this study is one of few to focus on the needs of bereaved carers, we recruited only eight bereaved carers, this area requires further focused attention.

We were unable to recruit health professionals to participate in focus groups as we recruited nationally across different time zones. However, 22 health professionals participated in semi-structured interviews, and we were able to achieve information power for this participant group.

### 4.2. Implications for Practice

This study describes the information needs of people with sarcoma, their carers, and HCPs (See Figure 1 for summary). Our findings have informed the online information resources we are currently developing for our sarcoma website (SUN-SHINE–**S**arcoma **U**nmet **N**eeds—**Sh**ared design of **In**formation and **E**ducation resources). As sarcoma affects children and adults, we are developing separate resources for different age groups, carers, family members, and bereaved carers to address information needs and provide channels for connection.

## 5. Conclusions

The needs of people with sarcoma and their carers are complex and change across the disease and survivorship trajectory. Information is often sought online, but is jargon-heavy or lacks relevance for those living in Australia. While HCPs were a helpful source of information, systemic issues limited information provided. People with sarcoma and their carers desire higher-quality online information to reduce anxiety and foster empowerment during their management of the complexities of a rare cancer. Future work should develop these tailored resources and cater to information and supportive care needs.

## Figures and Tables

**Table 1:** Patient, Carers and Bereaved Carers Demographic Data (n = 37).

	People with Sarcoma (n = 18)	Carers (n = 11)	Bereaved Carers (n = 8)
**Age at interview (years)**			
Mdn (IQR)	46.4 (15.3)	53.0 (7.0)	48.8 (13.8)
Min.-Max.	18–70	43–65	22–64
**Age at diagnosis (years) for person with sarcoma who was participating or being cared for**	n (%)	n (%)	n (%)
Adolescent (10–19)	3 (16.7)	9 (81.8)	6 (75)
Young adult (20–24)	0 (0)	0 (0)	1 (12.5)
Adult (25–40)	4 (22.2)	0 (0)	1 (12.5)
Adult (>40)	11 (61.1)	2 (18.2)	0 (0)
**Sex**			
Female	13 (72.2)	10 (90.9)	8 (100)
Male	5 (27.8)	1 (9.1)	0 (0)
**State**			
WA	8 (44.4)	6 (54.5)	1 (12.5)
QLD	4 (22.2)	1 (9.1)	0 (0)
NSW	3 (16.7)	4 (36.4)	1 (12.5)
VIC	1 (5.6)	0 (0)	4 (50)
SA	1 (5.6)	0 (0)	0 (0)
TAS	1 (5.6)	0 (0)	0 (0)
ACT	0 (0)	0 (0)	2 (25)
**Location**			
Major city	11 (61.1)	8 (72.7)	7 (87.5)
Regional/rural	7 (38.9)	3 (27.3)	1 (12.5)
**Sarcoma type**			
Bone	5 (27.8)	7 (63.6)	6 (75)
Soft tissue	12 (66.7)	3 (27.3)	2 (25)
Unspecified/unknown	1 (5.6)	1 (9.1)	0 (0)
**Carer role**			
Parent	-	9 (81.8)	5 (62.5)
Spouse	-	2 (18.2)	1 (12.5)
Sister	-	0 (0)	1 (12.5)
Family friend	-	0 (0)	1 (12.5)

**Table 2:** Healthcare Professionals Demographic Data (n = 22).

HCPs (n = 22)	n (%)
**Sex**	
Female	16 (72.7)
Male	6 (27.3)
**State**	
WA	12 (54.5)
NSW	3 (13.6)
ACT	2 (9.1)
SA	2 (9.1)
VIC	2 (9.1)
QLD	1 (4.5)
Location	
Major city	22 (100)
**Profession**	
Nurse	7 (31.8)
Psychologist/Counsellor/Youth Worker	4 (18.2)
Oncologist	3 (13.6)
Care Coordinator	2 (9.1)
Exercise Physiologist	2 (9.1)
Occupational Therapist	1 (4.5)
Physiotherapist	1 (4.5)
Surgeon	1 (4.5)
Sarcoma researcher	1 (4.5)
Oncologist	3 (13.6)
Care Coordinator	2 (9.1)

## Data Availability

The data presented in this study are available on request from the corresponding author due to ethical reasons.

## Abbreviations

The following abbreviations are used in this manuscript:

PwS	Person with sarcoma
C	Informal carer of person with sarcoma
BC	Bereaved Carer of person with sarcoma
HCP	Healthcare professional
